# Factors triggering familial mediterranean fever attacks, do they really exist?

**DOI:** 10.1007/s11739-024-03576-w

**Published:** 2024-03-15

**Authors:** Kerem Parlar, Muhammed Bahaddin Ates, Mehmet Erinc Onal, Ece Bostancı, Feyza Nur Azman, Serdal Uğurlu

**Affiliations:** 1 Cerrahpasa Medical Faculty, Istanbul University-Cerrahpasa, Istanbul, Turkey; 2 Division of Rheumatology, Department of Internal Medicine, Cerrahpasa Medical Faculty, Istanbul University-Cerrahpasa, 53 Kocamustafapasa Street, Fatih, Istanbul, 34098 Turkey

**Keywords:** Familial Mediterranean fever, Stress, Fatigue, Colchicine

## Abstract

Several possible factors are hypothesized to trigger familial Mediterranean fever (FMF) attacks; however, there is no consensus on this matter. We aimed to identify these triggering factors and analyze their relationship with the Mediterranean fever gene mutation status. We prepared a questionnaire that included the most commonly mentioned possible trigger factors of familial Mediterranean fever. We administered a questionnaire to 882 patients. We used a questionnaire assessing the following: psychological stress, consumption of tea and coffee, relationship with menses, menopause and post-menopausal alleviation, seasonal changes, traveling for long durations, relocation, starvation, sleeplessness, cold exposure, fatigue, wind exposure, and humidity. The most frequent triggering factor for familial Mediterranean fever attacks was psychological stress (75.2%). Cold exposure was a statistically significant trigger in patients with exon 10 mutations. Humidity was a statistically significant trigger in patients with exon 2 mutations. Seasonal changes, traveling for long durations, relocation, and cold exposure were statistically significant triggers of familial Mediterranean fever attacks in patients with homozygous M694V mutations. Identifying trigger factors can lead to better preventive measures and personalized therapies to decrease familial Mediterranean fever attacks. Patients can significantly decrease the number of familial Mediterranean fever attacks they experience by managing psychological stress and avoiding physical factors such as cold exposure and fatigue. Determining the relationship between trigger factors and patients' Mediterranean fever gene mutation status can lead to personalized therapy for the prevention of familial Mediterranean fever attacks.

**Supplementary Information:**

The online version contains supplementary material available at 10.1007/s11739-024-03576-w.

## Introduction

Autoinflammatory diseases are characterized by recurrent inflammation and fever, without an underlying source of infection or autoimmune process [1]. Familial Mediterranean fever (FMF) is a prototypic hereditary autoinflammatory disease characterized by recurrent febrile attacks accompanied by peritonitis, synovitis, pleuritis, and pericarditis [2]. The Mediterranean fever (MEFV) gene, located on the short arm of chromosome 16, is mutated in patients with FMF. MEFV gene encodes pyrin protein, which is an essential component of the NLRP3 inflammasome complex [3, 4]. Pathogenic MEFV mutations lead to gain-of-function mutations in pyrin protein [5]. Caspase-1 activation and IL-1β production due to mutations in pyrin protein causes excessive inflammation [4]. Systemic amyloidosis is the feared complication of FMF as it results in increased morbidity and mortality [6].

Colchicine is the primary medication used to treat FMF. It binds to β-tubulin and disrupts mitotic spindle formation. Colchicine also activates Ras homologous guanosine triphosphatase (Rho GTPase), thereby suppressing pyrin activity [5]. The prophylactic use of colchicine prevents FMF attacks and amyloidosis development [7]. However, in some patients, the disease is resistant to colchicine, or colchicine is not well-tolerated. In such cases, IL-1 inhibitors such as anakinra and canakinumab are the preferred options for treatment [8, 9]. The main goal of FMF treatment is to prevent febrile attacks however it is impossible to know the timing of an attack. Knowing the triggering factors of FMF attacks can help predict an upcoming attack and take preventative actions.

Several factors have been hypothesized to trigger an FMF attack. Possible triggering factors include emotional stress, menstruation, and cold exposure. However, the number of studies in this area is limited. Furthermore, the relationship between the MEFV mutation status and susceptibility to potential trigger factors remains a matter of curiosity. In this study, we aimed to update earlier findings and enlighten these topics of curiosity with newly obtained data. We surveyed a group of FMF patients to discover potential triggers of FMF attacks and their relationship with MEFV mutation status.

## Methods

In this study, we enrolled 882 adult patients diagnosed with FMF according to the Tel-Hashomer criteria. These patients are currently being followed-up at our adult rheumatology clinic. The study was approved by the Istanbul University Cerrahpasa Ethical Committee (reference code:7LvSwj7h) and informed consent was obtained from all participants.

This study used a cross-sectional design. We prepared a questionnaire that included the most commonly mentioned possible trigger factors for FMF. We included the most commonly mentioned trigger factors in the literature and those most commonly mentioned by our patients. We used a questionnaire assessing the following: psychological stress, consumption of tea and coffee, relationship with menses, menopause and post-menopausal alleviation, seasonal changes, traveling for long durations, relocation, starvation, sleeplessness, cold exposure, fatigue, wind exposure, and humidity (Table 1). We also questioned attack characteristics such as fever, abdominal pain, chest pain, arthritis, arthralgia, myalgia, and erysipelas-like rash. Demographic characteristics, age at disease onset, age at diagnosis, and MEFV mutation status were obtained from medical records. MEFV mutation genetic analysis was performed by amplifying exons 2, 3, 5 and 10 via PCR.

**Table 1:** Triggering factors prior to the attacks

Factor	n = 882
Psychological stress	663 (75.2%)
Excessive tea/coffee consumption	61 (6.9%)
Seasonal changes	500 (56.7%)
Traveling for long durations	286 (32.4%)
Relocation	236 (26.8%)
Starvation	139 (15.8%)
Sleeplesness	334 (37.9%)
Cold exposure	440 (49.9%)
Fatigue	561 (63.6%)
Wind exposure	337 (38.2%)
Humidity	266 (30.2%)
Menstruation (n = 496)	253 (51.0%)

Descriptive statistics are presented as numbers and percentages for categorical variables. They are presented as mean ± standard deviation for numerical variables. Pearson’s chi-square test and Fisher’s exact test were used to compare categorical variables and Student’s t-test was used to compare means between two groups. Statistical significance was set at p < 0.05. All analyses were performed using the open-source R software v.4.1.2, running under IDE RStudio v.2022.07.1.

## Results

We administered the questionnaire to 882 patients with familial Mediterranean fever (FMF), 386 men and 496 women. The demographic and disease characteristics of patients are presented in Table 2. The mean age was 39.4 ± 11.7 years. The mean age at the onset of FMF symptoms was 16.2 ± 10.5 years, and the age at initial FMF diagnosis was 22.8 ± 13.0 years. The diagnostic delay was calculated to be 8.49 ± 10.6 years.

**Table 2:** Demographic and disease characteristics of patients

	n = 882
Gender, (male/female)	386/496
Age, years (mean ± SD)	39.4 ± 11.7
Age at disease onset, years (mean ± SD)	16.2 ± 10.5
Age at the diagnosis, years (mean ± SD)	22.8 ± 13.0
Diagnostic delay, years (mean ± SD)	8.49 ± 10.6
Characteristics of FMF attacks	
Attack duration, hours (mean ± SD)	71.3 ± 72.0
Fever, n (%)	606 (68.7)
Peritonitis, n (%)	739 (83.8)
Pleuritis and/or pericarditis, n (%)	490 (55.6)
Arhtritis, n (%)	397 (45.0)
Arthralgia, n (%)	656 (74.4)
Myalgia, n (%)	557 (63.2)
Erysipelas-like erythema, n (%)	178 (20.2)

The characteristics of the FMF attacks are displayed in Table 2. The most common characteristics were peritonitis (83.8%), arthralgia (74.4%), and fever (68.7%). The mean attack duration was 71.3 ± 72.0 h. In our cohort of 882 patients, 665 underwent genetic testing (75.4%) (Table 3). Of the 665 patients, 18.9% were homozygous for M694V, 80.0% had mutations located in exon 10, and 18.0% had mutations located in exon 2.

**Table 3:** MEFV mutation characteristics of FMF patients

Mutation test done, n (%)	665 (75.4)
M694V homozygous, n (%)	126 (18.9)
M694V heterozygous, n (%)	263 (39.5)
M680I homozygous, n (%)	23 (3.5)
M680I heterozygous, n (%)	97 (14.6)
V726A homozygous, n (%)	12 (1.8)
V726A heterozygous, n (%)	90 (13.5)
M694I homozygous, n (%)	0
M694I heterozygous, n (%)	3 (0.5)
R761H homozygous, n (%)	1 (0.2)
R761H heterozygous, n (%)	24 (3.6)
E148Q homozygous, n (%)	3 (0.5)
E148Q heterozygous, n (%)	70 (10.5)
R202Q homozygous, n (%)	7 (1.1)
R202Q heterozygous, n (%)	45 (6.8)
Other MEFV mutation, n (%)	39 (5.9)
Negative MEFV mutation, n (%)	49 (7.4)
Single heterozygous, n (%)	238 (35.8)
Compound heterozygous, n (%)	155 (23.3)
Exon 10 mutation, n (%)	532 (80.0)
Exon 2 mutation, n (%)	120 (18.0)

Table 1 presents the trigger factors asked in the questionnaire. Ninety-four percent of the patients reported the presence of at least one triggering factor for their FMF attacks, with only 53 patients not reporting any triggering factor. The majority of patients reported the presence of more than one trigger factor, with 746 patients (84.6%) reporting the presence of two or more trigger factors and 83 patients (9.4%) reporting the presence of exactly one trigger factor. The most common trigger for FMF was psychological stress (75.2%). Fatigue (63.6%) was the second-most common trigger factor associated with FMF attacks.

Seasonal changes was also a common trigger, with 56.7% of patients reporting a worsened or increased frequency of attacks in response to seasonal variations. Cold exposure (49.9%) was also a notable trigger for FMF attacks. Starvation (15.8%) and excessive consumption of tea or coffee (6.9%) were other notable trigger factors. However, five patients (0.56%) reported alleviation of attack symptoms with excessive tea or coffee consumption.

Among the 496 female patients, 51.0% reported an increase in the frequency and intensity of attacks during menstruation. Menopause affected 21.4% of the female patients, and 51 patients (48.5%) reported a reduction in the frequency and intensity of attacks after entering menopause.

The relationship between possible trigger factors and the genetic test results of the patients was also analyzed. The analysis was divided into three groups based on mutations: homozygous M694V mutations, exon 10, and exon 2 mutations.

Cold exposure triggered FMF attacks in 52.63% of the patients with exon 10 mutation (Table 4). The relationship between cold exposure triggering FMF attacks and exon 10 mutation was statistically significant (p = 0.044).

**Table 4:** Relationship between the triggering factors and mutations in exon 10

Factor	Exon 10 mutation present (n = 532)	Exon 10 mutation not present (n = 133)	p value
Psychological stress	399 (75.0%)	98 (73.7%)	0.755
Excessive tea/coffee consumption	38 (7.1%)	7 (5.3%)	0.440
Seasonal changes	300 (56.4%)	72 (54.1%)	0.639
Traveling for long durations	178 (33.5%)	33 (24.8%)	0.055
Relocation	139 (26.1%)	35 (26.3%)	0.965
Starvation	87 (16.4%)	27 (20.3%)	0.280
Sleeplesness	202 (38.0%)	58 (43.6%)	0.233
Cold exposure	280 (52.3%)	57 (42.9%)	**0.044**
Fatigue	346 (65.0%)	82 (61.7%)	0.466
Wind exposure	210 (39.5%)	54 (40.6%)	0.812
Humidity	156 (29.3%)	47 (35.3%)	0.178
Menstruation	156 (29.3%)	37 (27.8%)	0.272
Attack duration, h (mean ± SD)	71.4 ± 38.5	73.2 ± 58.1	0.732

Bold value is statistically significant (p < 0.05)

Humidity (p = 0.023) as a trigger of FMF attacks were associated with exon 2 mutation. Exon 2 mutation also had a statistically significant relationship with attack duration (p = 0.015), as patients with exon 2 mutations had longer durations of attacks.

Seasonal changes (p = 0.036), traveling for long durations (p < 0.001), relocation (p = 0.002), and cold exposure (p = 0.016) were statistically significant triggers of FMF attacks among patients who were homozygous for M694V (Table 5).

**Table 5:** Relationship between the triggering factors and M694V homozygous mutations

Factor	M694V homozygous (n = 126)	Not M694V homozygous (n = 539)	p value
Psychological stress	99 (78.6%)	398 (73.8%)	0.271
Excessive tea/coffee consumption	13 (10.3%)	32 (6.9%)	0.078
Seasonal changes	81 (64.3%)	291 (54.0%)	**0.036**
Traveling for long durations	58 (46.0%)	153 (28.4%)	**< 0.001**
Relocation	47 (37.3%)	127 (23.6%)	**0.002**
Starvation	27 (21.4%)	87 (16.1%)	0.156
Sleeplesness	49 (38.9%)	211 (39.1%)	0.957
Cold exposure	76 (60.3%)	261 (48.4%)	**0.016**
Fatigue	87 (69.0%)	341 (63.3%)	0.222
Wind exposure	58 (46.0%)	206 (38.2%)	0.107
Humidity	42 (33.3%)	161 (29.9%)	0.447
Menstruation	36 (28.6%)	157 (29.1%)	0.177
Attack duration, h (mean ± SD)	73.3 ± 40.8	71.4 ± 43.7	0.720

Bold values are statistically significant (p < 0.05)

The relationship between triggering factors and drugs used in the treatment is shown in Table 6. There were no statistically significant associations regarding the triggering factors and treatment regimens.

**Table 6:** Relationship between the triggering factors and drugs used in treatment

Factor	Patients using anti-IL1-agents (n = 21)	Patients using colchicine (n = 697)	p value
Psychological stress	15 (71.4%)	536 (76.9%)	0.600
Excessive tea/coffee consumption	0 (0%)	49 (7.0%)	0.389
Seasonal changes	12 (57.1%)	387 (55.5%)	1.000
Traveling for long durations	7 (33.3%)	249 (35.7%)	0.816
Relocation	5 (23.8%)	188 (27.0%)	1.000
Starvation	3 (14.2%)	112 (16.1%)	1.000
Sleeplesness	5 (23.8%)	267 (38.3%)	0.253
Cold exposure	11 (52.4%)	353 (50.6%)	1.000
Fatigue	12 (57.1%)	447 (64.1%)	0.499
Wind exposure	8 (38.0%)	276 (39.6%)	1.000
Humidity	8 (38.0%)	213 (30.6%)	0.476
Menstruation	5 (23.8%)	200 (28.7%)	0.807
Attack duration, h (mean ± SD)	61.7 ± 26.6	71.1 ± 38.5	0.367

## Discussion

In this study, we surveyed a group of FMF patients regarding the factors triggering their attacks and analyzed their relationship with the patient’s MEFV mutation status. This study has the largest patient population (n = 882) among the studies that examined the triggering factors of FMF attacks. When we look at the historical perspectives of perception of triggers in FMF, the first well-established epidemiologic study about triggering factors of FMF attacks was conducted by Yenokyan et al. [10]. They reported an association between the number of experienced stressful events and FMF attacks. They suspected that an inappropriate response of the hypothalamic–pituitary–adrenal axis to stress may have caused this association. Despite the late conduction of the first formal epidemiologic study, reports on triggers in FMF are much older. The first reported psychosocial correlation of FMF attack incidence in children was reported by Gidron et al. [11]. One of the oldest reports on triggers in FMF was by Golden et al. [12]. The authors reported an association between menses and FMF attacks. Subsequent studies about menses being a trigger for FMF attacks in women were published almost three decades later by Ehrenfeld et al. [13], Ben-Chetrit et al. [14], Akar et al. [15], and Guzelant et al. [16]. Several other studies have been conducted on specific trigger factors such as stress exposure [17, 18], diet [19], and seasonal changes [20].

FMF can be challenging to diagnose, especially in geographical regions where it is not prevalent. It can present with different clinical phenotypes, symptoms can be non-specific, and diagnosis is based on clinical symptoms. Diagnostic delays and misdiagnoses are relatively common even in regions where the disease is prevalent. It was commonly misdiagnosed as acute appendicitis or acute rheumatic fever in a study by Erdogan et al. [21]. There are diagnostic scores that try to predict the MEFV mutation carrier status for adult patients presenting with periodic fever to better diagnose late-onset cases [22]. Trigger factors can also aid in diagnosing late-onset and atypical cases if they can be clearly defined with further studies in this field. FMF can also cause chronic organ damage if treatment is not initiated correctly. There are even scores, such as the Autoinflammatory Disease Damage Index (ADDI), which evaluates the degree of chronic damage. Knowing the trigger factors can lead to better disease control which will in turn lead to decreased chronic organ damage [23].

Karadag et al. [24] reported on the frequency of many potential triggering factors of FMF attacks such as cold exposure, psychological stress, tiredness, menstruation, sleeplessness, infection, and trauma which might trigger either serositis-dominated attacks or musculoskeletal pain-dominated attacks. A total of 275 patients were included in this study. In their study, cold exposure (59.3%) was the most common trigger factor. However, psychological stress (75.2%), fatigue (63.6%), seasonal changes (56.7%), and menstruation (51.0%) were more common than cold exposure (49.9%) in our study. They also analyzed the association between these potential triggers and individual mutations. They reported a statistically significant relationship (p < 0.032) between starvation and patients with M694V allele. Starvation was also included in our survey; however, it was infrequently reported (15.8%) and had no statistically significant association with a mutation. They also reported a few other statistically significant results between mutations and factors that trigger FMF attacks.

Kishida et al. [25] reported on the frequency of triggers of FMF attacks in Japanese patients. Their study included 372 patients and menstruation (39.7%) was the most common triggering factor for FMF attacks. Psychological stress and tiredness were second and third, respectively. These three factors were also among the most commonly encountered trigger factors in our study. While environmental change and cold exposure were among the most prevalent triggers of FMF attacks in our study, they were rarely mentioned in the study by Kishida et al. Environmental change have been reported to trigger FMF attacks in less than 5% of their patients, while cold exposure was found to trigger FMF attacks in less than 2% of patients. The dramatic difference in prevalence may be attributed to genetic, cultural, and geographical differences among the patient populations. Studies examining the effects of potential triggering events in diverse patient populations should be conducted to determine whether ethnicity or geographical variation plays a role in this association. This difference may also be attributed to the different MEFV mutation characteristics of the two patient populations. E148Q was the most common MEFV mutation in the study by Kishida et al., whereas M694V was the most frequent mutation in our patient population.

Psychological stress, menstruation, and fatigue were among the most common trigger factors in our study and the studies by Karadag et al. [24] and Kishida et al. [25]. Yenokyan et al. [10] also reported a statistically significant association between the number of experienced stressful events and FMF attacks. We suggest the conduction of prospective and more objective studies involving these three factors to further establish their relationship with FMF attacks. Decreasing psychological stress by either lifestyle changes or pharmacotherapy can lead to a significant decrease in FMF attacks, which will lead to an increase in the quality of life and life expectancy of patients with FMF. Patients can also decrease their overall fatigue levels by paying special attention to some aspects of their lifestyle. The fatigue levels of FMF patients aged between 13 and years were examined in a study by Sarac et al. [26]. Patients who engaged in more than 2560 MET-min/week of physical activity and slept for more than seven hours a day had significantly lower fatigue levels. Therefore, patients who experience fatigue as a trigger for their attacks can be advised to increase their activity levels and pay more attention to their sleep levels to decrease attack frequency.

The triggers of FMF attacks in adult patients were studied using a retrospective data-collection method. The main limitations of this study were the dependence on the patients’ recall ability and their subjective definition of an FMF attack. In addition, the questionnaire was administered only once, which may have led to an increased presence of recall bias in our study. A prospective design would allow us to determine additional trigger factors and their correlation with the magnitude of triggered attacks. It would also yield more reliable results, as the clinician can determine if the patient is actually experiencing an FMF attack. However, additional studies on this subject should be performed with better and more precisely defined trigger factors, as inadequate definition of trigger factors is a limitation of our study. The temporal relationship between the triggering factors and FMF attacks should also be clearly defined. Studies by Yenokyan et al. [10] and Farisogullari et al. [27] are good examples of studies in which both trigger factors and the temporal relationship between trigger factors and attacks are precisely defined. The questionnaires implemented in these studies can be used as examples when planning further studies.

It should also be noted that the data presented in Table 6 can’t be used to assess the relationship between triggering factors and drugs used in the treatment. This is because triggering factors were reported retrospectively, and the drugs used in the treatment were the ones that the patients were currently using at the time. However, this may give an idea regarding the relationship between triggering factors and disease severity, as patients who need to use anti-IL-1 agents generally have more severe disease that cannot be managed by colchicine alone. The presence of triggering factors in relation to colchicine resistance status was analyzed in a study by Farisogullari et al. [27]. They reported a significantly higher frequency of triggering factors in the colchicine-resistant group than in the colchicine-responsive group, and the percentage of attacks triggered by triggering factors decreased significantly in the colchicine-resistant group after anti-IL-1 agents were started. Another limitation of our study was that we did not analyze the association between comorbidities and triggering factors.

In conclusion, this study shows the high prevalence of triggering factors among patients with FMF. Advancements in this area have the potential to alter the way we manage patients with FMF, as it can provide new ways to prevent FMF attacks. This can result in increased implementation of preventive measures in the management of FMF. Psychological stress and fatigue were the most common triggers of FMF attacks among our patients. Patients can significantly decrease the number of FMF attacks they experience by managing psychological stress and avoiding physical factors such as cold exposure and fatigue. Determining trigger factors and their relationship with patient-specific factors such as mutation status and ethnicity can result in personalized treatment strategies for the prevention of FMF attacks. Developments in this area will lead to a decrease in the number of attacks and improved disease control.

### Supplementary Information

Below is the link to the electronic supplementary material.

Supplementary file1 (DOCX 16 KB)

## Data Availability

All data relevant to the study are included in the article.
