# Supplementary material for: Factors triggering familial mediterranean fever attacks, do they really exist?
Source: Intern Emerg Med. 2024 Mar 15;19(4):1007–13. doi: 10.1007/s11739-024-03576-w (PMC11186929; doi:10.1007/s11739-024-03576-w)
Supplement: Supplementary file 1 — Supplementary file1 (DOCX 16 KB) [file 11739_2024_3576_MOESM1_ESM.docx]

FMF trigger factors questionnaire (translated from Turkish)

- Does emotional stress trigger your FMF attacks? This can include sadness, grief, anxiety and feeling under pressure.
- Does consuming more than two cups of tea or coffee trigger your FMF attacks?
- Does seasonal changes trigger your FMF attacks? For example going from summer to fall/ fall to winter/ winter to spring/ spring to summer.
- Does traveling for long durations (>4 hours) in a transportation vehicle such as car, plane or bus trigger your FMF attacks.
- Does relocating/being present to/in a different city or country trigger your FMF attacks?
- Does starvation trigger your FMF attacks? For example when you didn’t eat any food for considerably longer than your regular intervals and were experiencing discomfort related to hunger.
- Does sleeplesness trigger your FMF attacks? This can both include staying up till late night and having an insufficient amount of sleep.
- Does feeling cold trigger your FMF attacks?
- Does fatigue trigger your FMF attacks? This can include feeling physically or mentally tired.
- Does being exposed to wind trigger your FMF attacks? For example being exposed to windy weather or ventilator.
- Does humidity trigger your FMF attacks?
- Does menstruation trigger your FMF attacks?
- Did you enter menopause? Did the frequency of your FMF attacks decrease after entering menopause?
